# An open RNA-Seq data analysis pipeline tutorial with an example of reprocessing data from a recent Zika virus study

**DOI:** 10.12688/f1000research.9110.1

**Published:** 2016-07-05

**Authors:** Zichen Wang, Avi Ma'ayan

**Affiliations:** 1 Department of Pharmacology and Systems Therapeutics, Icahn School of Medicine at Mount Sinai, New York, NY, Box 1603, USA; 2 BD2K-LINCS Data Coordination and Integration Center, Icahn School of Medicine at Mount Sinai, New York, NY, Box 1603, USA; 3 Mount Sinai Knowledge Management Center for Illuminating the Druggable Genome, Icahn School of Medicine at Mount Sinai, New York, NY, Box 1603, USA

**Keywords:** Systems biology, bioinformatics pipeline, gene expression analysis, RNA-seq

## Abstract

RNA-seq analysis is becoming a standard method for global gene expression profiling. However, open and standard pipelines to perform RNA-seq analysis by non-experts remain challenging due to the large size of the raw data files and the hardware requirements for running the alignment step. Here we introduce a reproducible open source RNA-seq pipeline delivered as an IPython notebook and a Docker image. The pipeline uses state-of-the-art tools and can run on various platforms with minimal configuration overhead. The pipeline enables the extraction of knowledge from typical RNA-seq studies by generating interactive principal component analysis (PCA) and hierarchical clustering (HC) plots, performing enrichment analyses against over 90 gene set libraries, and obtaining lists of small molecules that are predicted to either mimic or reverse the observed changes in mRNA expression. We apply the pipeline to a recently published RNA-seq dataset collected from human neuronal progenitors infected with the Zika virus (ZIKV). In addition to confirming the presence of cell cycle genes among the genes that are downregulated by ZIKV, our analysis uncovers significant overlap with upregulated genes that when knocked out in mice induce defects in brain morphology. This result potentially points to the molecular processes associated with the microcephaly phenotype observed in newborns from pregnant mothers infected with the virus. In addition, our analysis predicts small molecules that can either mimic or reverse the expression changes induced by ZIKV. The IPython notebook and Docker image are freely available at: http://nbviewer.jupyter.org/github/maayanlab/Zika-RNAseq-Pipeline/blob/master/Zika.ipynb and https://hub.docker.com/r/maayanlab/zika/.

## Introduction

The increase in awareness about the irreproducibility of scientific research requires the development of methods that make experimental and computational protocols easily repeatable and transparent ^1^. The advent of interactive notebooks for data analysis pipelines significantly enhances the recording and sharing of data, source code, and figures ^2^. In a subset of recent publications, an interactive notebook was published alongside customary manuscripts ^3^. Similarly, here we present an interactive IPython notebook ( http://nbviewer.jupyter.org/github/maayanlab/Zika-RNAseq-Pipeline/blob/master/Zika.ipynb) that serves as a tutorial for performing a standard RNA-seq pipeline. The IPython notebook pipeline provides scripts ( http://dx.doi.org/10.5281/zenodo.56311) that process the raw data into interactive figures and permits other downstream analyses that can enable others to quickly and properly repeat our analysis as well as extract knowledge from their own data. As an example, we applied the pipeline to RNA-seq data from a recent publication where human induced pluripotent stem cells were differentiated to neuronal progenitors and then infected with Zika virus (ZIKV) ^4^. The aim of the study was to begin to understand the molecular mechanisms that induce the observed devastating phenotype of newborn-microcephaly from pregnant mothers infected with the virus.

## Methods and results

**Figure 1.**
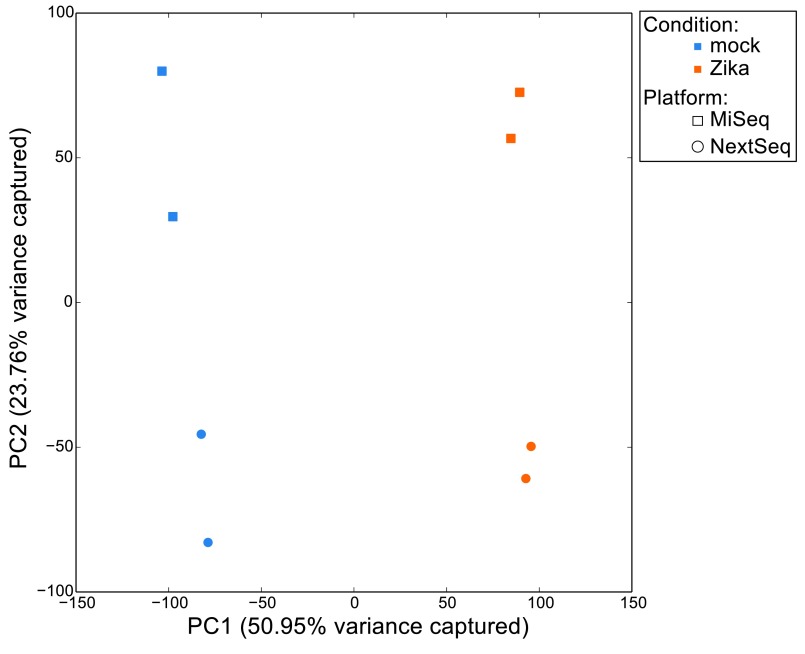
Principal Component Analysis (PCA) of the samples in the first two principal component space. ZIKV-infected and mock-treated cells are colored in orange and blue, respectively. The shapes of the dots indicate the sequencing platforms: MiSeq – squares, and NextSeq - circles.

**Figure 2.**
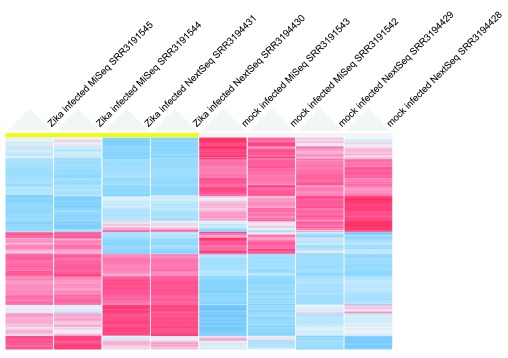
Hierarchical clustering heatmap of the 800 genes with the largest variance. The CPM of 800 genes with the largest variance across the eight samples were log transformed and z-score normalized across samples. Blue indicates low expression and red high.

The first publicly available study profiling gene expression changes after ZIKV infection of human cells was deposited into NCBI's Gene Expression Omnibus (GEO) in March 2016. The raw data is available ( ftp://ftp-trace.ncbi.nlm.nih.gov/sra/sra-instant/reads/ByStudy/sra/SRP/SRP070/SRP070895/) from the Sequence Read Archive (SRA) with accession number GSE78711. In this study, gene expression was measured by RNA-seq using two platforms: MiSeq and NextSeq ^4^ in duplicates. The total number of samples is eight, with four untreated samples and four infected samples. We first downloaded the raw sequencing files from SRA and then converted them to FASTQ files. Quality Control (QC) for the RNA-Seq reads was assessed using FastQC ^5^. The reports generated by FastQC were in HTML format and can be accessed through hyperlinks from the IPython notebook. The reads in the FASTQ files were aligned to the human genome with Spliced Transcripts Alignment to a Reference (STAR) ^6^. STAR is a leading aligner that accomplishes the alignment step faster and more accurately than other current alternatives ^6^. We next applied featureCounts ^7^ to assign reads to genes, and then applied the edgeR Bioconductor package ^8^ to compute counts per million (CPM) and reads per kilobase million (RPKM). The next steps are performed in Python within the IPython notebook. We first filtered out genes that are not expressed or lowly expressed. Subsequently, we performed principal component analysis (PCA) ( Figure 1). The PCA plots show that the samples cluster by infected vs. control cells, but also by platform. Next, we visualized the 800 genes with the largest variance using an interactive hierarchical clustering (HC) plot ( Figure 2). This analysis separates the groups of genes that are differentially expressed by infected vs. control from those that are differential by platform. The visualization of the clusters is implemented with an interactive external web-based data visualization tool called clustergrammer ( http://amp.pharm.mssm.edu/clustergrammer/). Clustergrammer provides interactive searching, sorting and zooming capabilities.

**Figure 3.**
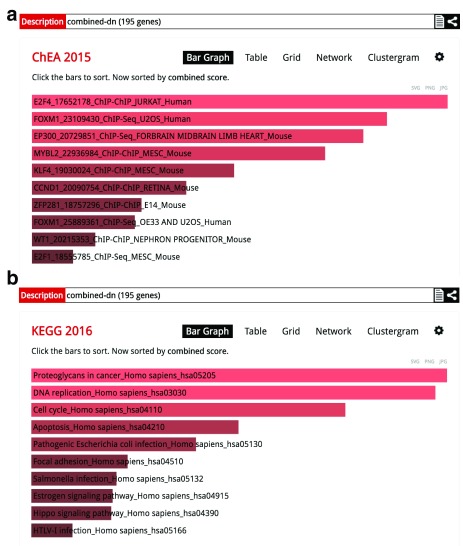
Bar plots of the top enriched gene sets from the ( **a**) ChEA and ( **b**) KEGG libraries for the downregulated genes after ZIKV infection.

The following step is to identify the differentially expressed genes (DEG) between the two conditions. This is achieved with a unique method we developed called the Characteristic Direction (CD) ^9^. The CD method is a multivariate method that we have previously demonstrated to outperform other leading methods that compute differential expression between two conditions ^9^. Once we have ranked the lists of DEG, we submit these for signature analysis using two tools: Enrichr ^10^ and L1000CDS2 ^11^. Enrichr queries the up and down gene sets against over 180,000 annotated gene sets belonging to 90 gene set libraries covering pathway databases, ontologies, disease databases, and more ^10^. The results from this enrichment analysis confirm that the downregulated genes after ZIKV infection are enriched for genes involved in cell cycle-related processes ( Figure 3a). These genes are enriched for targets of the transcription factors E2F4 and FOXM1 ( Figure 3b). Both transcription factors are known to regulate cell proliferation and play central role in many cancers. The downregulation of cell cycle genes was already reported in the original publication; nevertheless, we obtained more interesting results for the enriched terms that appeared most significant for the upregulated genes. Particularly, the top two terms from the mouse genome informatics (MGI) Mammalian Phenotype Level 4 library are abnormal nervous system (MP0003861) and abnormal brain morphology (MP0002152) ( Table S1). This library associates gene knockouts in mice with mammalian phenotypes. These enriched terms enlist a short set of genes that potentially link ZIKV infection with the concerning observed microcephaly phenotype. Finally, to identify small molecules that can potentially either reverse or mimic ZIKV-induced gene expression changes, we query the ZIKV-induced signatures against the LINCS L1000 data. For this, we utilize L1000CDS2 ^11^, a search engine that prioritize small molecules given a gene expression signature as input. L1000CDS2 contains 30,000 significant signatures that were processed from the LINCS L1000 data with the CD method. The results suggest small molecules that could be tested in follow-up studies in human cells for potential efficacy against ZIKV ( Table S2).

To ensure the reproducibility of the computational environment used for the whole RNA-Seq pipeline, we packaged all the software components used in this tutorial, including the command line tools, R packages, and Python packages into a Docker image. This Docker image is made publically available at https://hub.docker.com/r/maayanlab/zika/. The Docker image was created based on the specifications outlined on the official IPython’s Scipy Stack image ( https://hub.docker.com/r/ipython/scipystack/). The additional command line tools, R scripts, and Python packages together with their dependencies were compiled and installed into the Docker image. The RNA-Seq pipeline Docker image was deployed onto our Mesos cluster, which allows users to run the IPython notebook interactively. The Docker image can also be downloaded and executed on local computers and servers, or deployed in the cloud if users have access to cloud provider services with a Docker Toolbox installed ( https://www.docker.com/products/docker-toolbox). We also provide detailed instructions on how to download and execute the Docker image ( https://hub.docker.com/r/maayanlab/zika/).

The ‘Dockerization’ of the RNA-Seq pipeline facilitates reproducibility of the pipeline at the software level because the Docker image ensures that all versions of the software components are consistent and static. Dockerization also helps users to handle the complex installation of many dependencies required for the computational pipeline. Moreover, the Docker image can be executed on a single computer, clusters/servers and on the cloud. The only limitation of having a Docker image is that it prevents users from adding or altering the various steps which require additional software components and packages. However, advanced users can build their own Docker images based on our initial image to customize it for their needs.

## Discussion and conclusions

**Figure 4.**
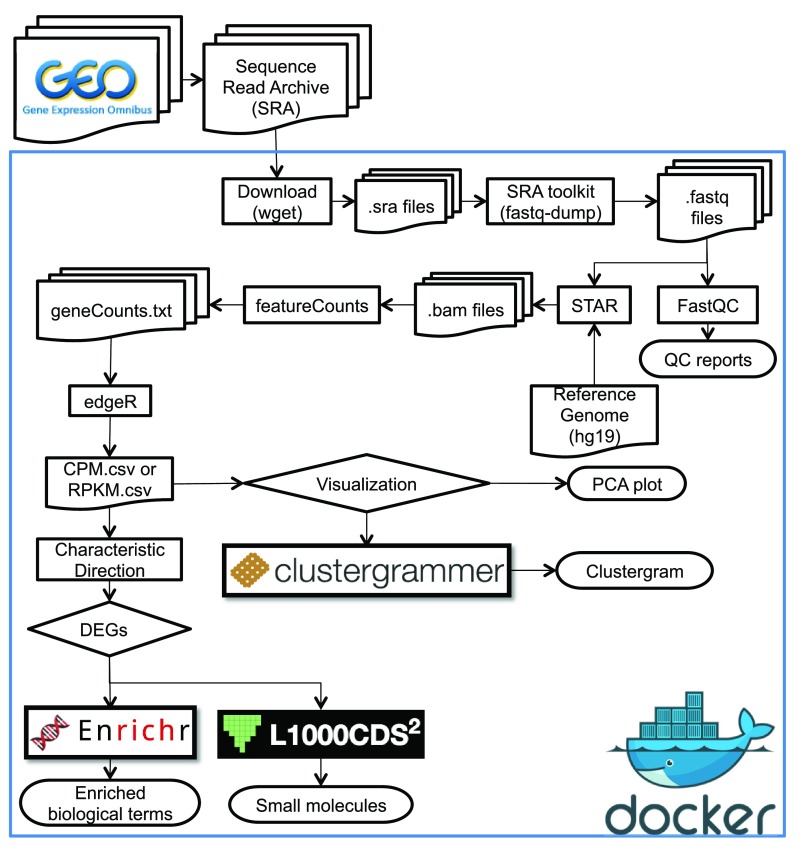
Workflow of the different steps carried out in the pipeline.

In summary, we provide an open source RNA-seq processing pipeline ( Figure 4) that can be used to extract knowledge from any study that profiled gene expression using RNA-seq applied to mammalian cells, comparing two conditions. The advantage of providing the pipeline in the IPython notebook format and as a Docker container is that it enables others to quickly reproduce our results with minimal overhead and potentially apply similar methodology for the analysis of other similar datasets. Advanced users can add, improve and customize the pipeline by forking it on GitHub. The results that we obtained for ZIKV are consistent with the results published in the original study, but also enhance those findings by discovering a link between the upregulated genes and genes that, when knocked out in mice, induce morphological brain defects. Some of these genes could be the causal genes of the microcephaly phenotype observed in newborns of mothers infected with the virus. Nevertheless, caution should be used when interpreting these results because they may simply indicate a reduction in cell cycle activity and an increase in neuronal differentiation of the type of cells used in the original study.

## Data and software availability

The IPython notebook, as well as other scripts and data files for this tutorial are available on GitHub at: https://github.com/MaayanLab/Zika-RNAseq-Pipeline, doi: http://dx.doi.org/10.5281/zenodo.56311 ^12^.

The Docker image for this tutorial is available on DockerHub at: https://hub.docker.com/r/maayanlab/zika/.

## Supplementary material

**Table S1.**

Top enriched gene sets from the MGI Mammalian Phenotype Level 4 gene set library for the upregulated genes after ZIKV infection.

**Table S2.**

(a) Top scoring small molecules that are potential mimickers of the ZIKV infection signatures. (b) Top scoring small molecules that are potential reversers of the ZIKV infection signatures.
