# Supplementary material for: An open RNA-Seq data analysis pipeline tutorial with an example of reprocessing data from a recent Zika virus study
Source: F1000Res. 2016 Jul 5;5:1574. [Version 1] doi: 10.12688/f1000research.9110.1 (PMC4972086; doi:10.12688/f1000research.9110.1)
Supplement: Peer review history

# Peer review: Referee response for version 1

**DOI:** 10.5256/f1000research.9804.r15289

**Published:** 2016-08-15

**Authors:** Fredrik Pettersson, Sarbashis Das

**Affiliations:** 1 Department of Cell and Molecular Biology, Uppsala University, Uppsala, Sweden

Wang and Ma’ayan have developed an open source RNAseq processing pipeline using standard methods as well as integrated tools for visualization and data analysis of differentially expressed genes between two sets of data. They test the pipeline by reanalyzing a previously published study of Zika virus infected human cells. They replicated the main result of the original study where genes related to the cell cycle were downregulated after infection. They also find a potential link to genes involved in brain morphology and a normal functioning of the nervous system in mice, something the original study missed. These genes are upregulated after Zika virus infection. This pipeline should be useful in any type of study where two conditions are compared, e.g. infected vs uninfected cells or treated vs untreated condition.

 Minor comments:

1. Figure 2. Have the different conditions/figure labels been mixed up? The 2 Zika infected MiSeq conditions look complementary to the 2 mock infected NextSeq controls, while the 2 Zika infected NextSeq conditions look complementary to the 2 mock infected MiSeq controls.
2. Figure 3a and 3b have been mixed up. 3b should be 3a and vice versa.

We have read this submission. We believe that we have an appropriate level of expertise to confirm that it is of an acceptable scientific standard.

# Peer review: Referee response for version 1

**DOI:** 10.5256/f1000research.9804.r15290

**Published:** 2016-07-29

**Authors:** Apostolos Zaravinos

**Affiliations:** 1 The School of Sciences, European University Cyprus, Engomi, Cyprus

The authors provide an open source RNA-seq processing pipeline that can be used to extract differential expression data between two conditions from an RNA-seq experiment. To test this, data from a recent publication where human iPSCs were differentiated to neuronal progenitors and then infected with Zika virus (ZIKV), were analysed with their pipeline, and their results were consistent with the original ones. Of interest, the authors discovered a link between the upregulated genes of this study and genes that, when knocked out could cause microcephaly observed in newborns of mothers infected with ZIKV. Overall, this is a novel and exciting computational protocol that promotes reproducibility and transparency of the results, and it is definately worth to be tested in other conditions, as well (eg, cancer).

 Minor comment: Perhaps its a matter of zoom-in/out of the clustergrammer, but it would be nice to show the names of the top up-/down-regulated genes (or their categories) as depicted in Figure 2.

I have read this submission. I believe that I have an appropriate level of expertise to confirm that it is of an acceptable scientific standard.

# Peer review: Referee response for version 1

**DOI:** 10.5256/f1000research.9804.r14924

**Published:** 2016-07-21

**Authors:** Ravi K Madduri

**Affiliations:** 1 Computation Institute, University of Chicago, Chicago, IL, USA

Wang and Ma’ayan introduced an RNA-seq pipeline tutorial using IPython notebook and a Docker image. Specifically, the authors applied the pipeline to analyze data from a recent Zika virus study. The authors found that their pipeline not only confirms the down-regulated cell cycle genes, but also uncovers a set of genes with a biological function that potentially associated with a particular phenotype. While the work and context sound interesting, there are several concerns that need to be addressed or discussed:

  

1. This reviewer really liked the approach that the authors have taken to showcase analysis. I wish more researchers adopt this methodology. 
2. This could be a great way to do additional analysis easily.. I wonder if authors can look into additional RNASeq pipelines and compare/contrast how Jupiter-friendly they are.  

Minor comments:

 The clustering (in Figure 1) is based on z-score and the 800 genes serve well to cluster the samples into two different groups. Was z-scores close to zero excluded as they are uninformative? Was FDR applied?

I have read this submission. I believe that I have an appropriate level of expertise to confirm that it is of an acceptable scientific standard.
